# Production of the PET radionuclide ^61^Cu via the ^62^Ni(p,2n)^61^Cu nuclear reaction

**DOI:** 10.1186/s41181-023-00233-z

**Published:** 2024-01-05

**Authors:** Santiago Andrés Brühlmann, Martin Walther, Klaus Kopka, Martin Kreller

**Affiliations:** 1 Institute of Radiopharmaceutical Cancer Research, Helmholtz-Zentrum Dresden-Rossendorf, Bautzner Landstraße 400, 01328 Dresden, Germany; 2 Faculty of Chemistry and Food Chemistry, School of Science, Technische Universität Dresden, 01062 Dresden, Germany; 3 National Center for Tumor Diseases (NCT) Dresden, University Hospital Carl Gustav Carus, Fetscherstraße 74, 01307 Dresden, Germany; 4 German Cancer Consortium (DKTK), Partner Site Dresden, Fetscherstraße 74, 01307 Dresden, Germany

**Keywords:** Copper-61, Targetry, Target chemistry, PET, Theranostics, Radiocopper, CopperNostics

## Abstract

**Background:**

There are only a handful of true theranostic matched pairs, and in particular the theranostic radiocopper trio ^61^Cu, ^64^Cu and ^67^Cu, for diagnosis and therapy respectively, is a very attractive candidate. In fact, the alternative of two imaging radionuclides with different half-lives is a clear advantage over other theranostic pairs, since it offers a better matching for the tracer biological and radionuclide physical half-lives. Due to the high availability of ^64^Cu, its translation into the clinic is being successfully carried out, giving the example of the FDA approved radiopharmaceutical Detectnet (copper Cu 64 dotatate injection). However, a shorter-lived PET radionuclide such as ^61^Cu may as well be beneficial.

**Results:**

Proton irradiation of enriched ^62^Ni electrodeposited targets with a compact cyclotron produced the desired radionuclide via the ^62^Ni(p,2n)^61^Cu nuclear reaction, leading to ^61^Cu activities of up to 20 GBq at end of bombardment and 8 GBq at end of purification. Furthermore, two purification methods are compared leading to comparable results regarding separation yield and product purity. Following the radiochemical separation, quality assessment of this product [^61^Cu]CuCl_2_ solution proved radionuclidic purities (RNP) over 99.6% and apparent molar activities (AMA) of 260 GBq/µmol with the 1,4,8,11-tetraazacyclotetradecane-1,4,8,11-tetraacetic acid (TETA) chelator, end of purification corrected.

**Conclusions:**

In the current article a comprehensive novel production method for the PET radionuclide ^61^Cu is presented, providing an alternative to the most popular production routes. Characterization of the [^61^Cu]CuCl_2_ product showed both high RNP as well as high AMA, proving that the produced activity presented high quality regarding radiolabeling up to 9 h after end of purification. Furthermore, production scalability could be easily achieved by increasing the irradiation time.

**Graphical abstract:**

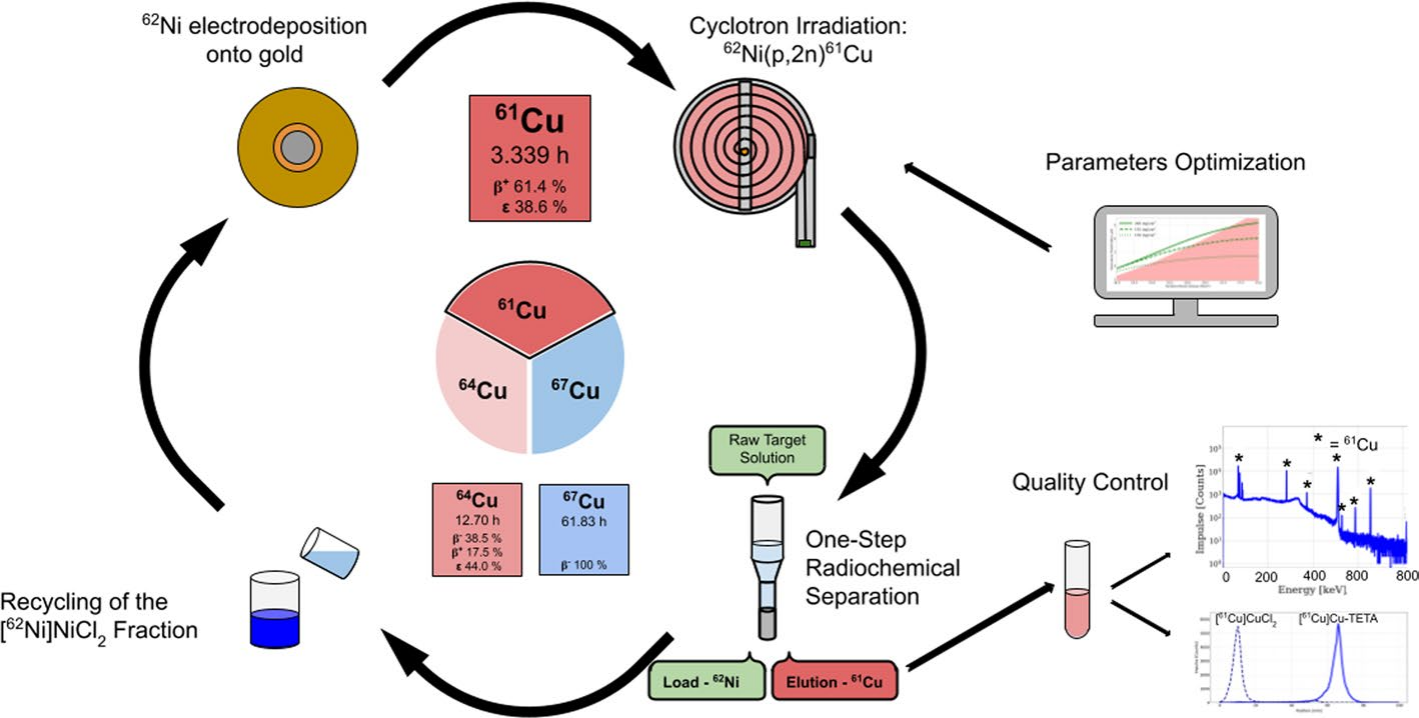

**Supplementary Information:**

The online version contains supplementary material available at 10.1186/s41181-023-00233-z.

## Background

During the last decades, Positron Emission Tomography (PET) has gained importance in cancer diagnosis. This technique in combination with other imaging modalities, i.e. computed tomography (PET/CT) and magnetic resonance imaging (PET/MRI), have proven to be a powerful diagnostic tool for tumor detection (Cherry et al. 2012; Schulthess and Schlemmer 2008). Although the most widely used radionuclide in PET is the non-metal ^18^F, e.g. as [^18^F]FDG, [^18^F]FDOPA, i.a. (Coenen et al. 2010), a radiometal can also be incorporated into a targeting vector. The radiometal ion can be complexed by a suitable chelator conjugated to a tracer molecule (Okoye et al. 2019). In particular this is the case of ^68^Ga-based radiopharmaceuticals, a positron-emitter with a half-life of approximately one hour. The high availability of this radionuclide due to its ^68^Ge/^68^ Ga generator-based production is of primordial importance (Velikyan 2014), however, with the advances in the accelerator-based production of radiometals, other metallic radionuclides show a huge potential. On the one hand, radionuclides with half-lives of only a couple of hours and thus comparable to ^68^Ga have been looked into. Some radionuclides from this group are ^43/44^Sc (half-lives 3.89 h and 3.97 h) (Domnanich et al. 2017; Becker et al. 2023; Chaple and Lapi 2018), ^45^Ti (3.08 h) (Chaple and Lapi 2018), ^61^Cu (3.34 h) (Szelecsényi et al. 1993; McCarthy et al. 1999; Thieme et al. 2013), and ^132/133^La (4.8 h and 3.91 h) (Aluicio-Sarduy et al. 2019; Nelson et al. 2022; Brühlmann et al. 2022), (properties presented in a comparative table in Additional file 1). On the other hand, radionuclides with half-lives between 9 and 20 h have been investigated, e.g. ^55^Co (17.53 h), ^64^Cu (12.7 h), ^66^Ga (9.3 h), ^86^Y (14.7 h), ^90^Nb (14.6 h), ^152^Tb (17.5 h) (Aluicio-Sarduy et al. 2018; Mikolajczak et al. 2019).

Only a couple of these β^+^-emitters have a therapeutic counterpart radionuclide forming a true theranostic matched pair. Such is the case of ^86^Y/^90^Y, ^43/44^Sc/^47^Sc, ^152^Tb/^149/161^Tb and ^61/64^Cu/^67^Cu matched radionuclides. While ^90^Y and ^161^Tb production is based on neutron reactions, i.e. nuclear reactors (Mikolajczak et al. 2019), radioscandium and radiocopper pairs can be entirely produced with cyclotrons, i.a. via proton or deuteron irradiation, or photonuclear reactions. Moreover, in the last couple of years ^64^Cu radiochemistry and radiopharmaceuticals have been well established (IAEA 2023), even with the FDA approved radiopharmaceutical Detectnet (copper Cu 64 dotatate injection) (Torre et al. 2020), while radioscandium isotopes are relegated on this matter.

The decay characteristics of copper radioisotopes potentially useful for nuclear medicine purposes are shown in Table 1 to illustrate their prospects (Hussain et al. 2023; IAEA 2023). What is more, recent advances on its therapeutic counterpart production, ^67^Cu, via several paths potentially leading to GBq quantities (Copper-67,2022; Nigron et al. 2021; Brühlmann et al. 2023), further motivates the study of copper-based radiopharmaceuticals.

**Table 1:** Physical properties of copper radionuclides of interest for nuclear medicine

Radionuclide	Half-life	E_β+,mean_/keV (Intensity/%)	E_β-,mean_/keV (Intensity/%)	E_γ_/keV (Intensity/%)
^60^Cu	23.7 m	872 (49)		1333 (88)
		1325 (15)		1792 (45.4)
		840 (11.6)	No emission	826.4 (21.7)
		1720 (5)		3124 (4.8)
		805 (4.6) i.a		1862 (4.8) i.a
		524 (51)		282.9 (12.7)
^61^Cu	3.34 h	399 (5.8)	No emission	656.0 (10.4)
		238 (2.5)		67.41 (4.0)
		494 (2.1)		1185 (3.6) i.a
^62^Cu	9.67 m	1321 (97.6)	No emission	1173 (0.34) i.a
^64^Cu	12.7 h	278 (17.5)	191 (38.5)	1346 (0.47)
^67^Cu	61.8 h	No emission	121 (57)	184.5 (48.7)
			154 (22)	93.3 (16.1)
			189 (20)	91.3 (7.9) i.a
			51 (1.1) i.a	

On the one hand, ^60^Cu and ^62^Cu have a high β^+^ energy emission that impacts negatively the quality of the PET scan, however, their short half-lives motivate their use for small molecules and low-molecular weight molecules, with fast pharmacokinetics, i.a. as perfusion and hypoxia tracers. On the other hand, the longer-lived ^64^Cu results are attractive for labeling of peptides, peptidomimetics or antibodies (Williams et al. 2005). Moreover, ^61^Cu comes to fill the gap between the short-lived ^60/62^Cu and ^64^Cu. In fact, ^61^Cu is an interesting alternative to ^64^Cu, with a shorter half-life and higher β^+^ particle emission yield, but also a higher β^+^ energy (Williams et al. 2005; Rowshanfarzad et al. 2006). Furthermore, ^61^Cu showed some advantages over ^64^Cu for the tracking of small proteins regarding dosimetry concerns, due to the latter’s longer half-life and its β^−^ co-emission with 38.5% intensity (McCarthy et al. 1999; Zhang et al. 2012).

Different paths have been proposed for the production of ^61^Cu, from nickel and zinc targets. Promising results were obtained via the ^60^Ni(d,n)^61^Cu nuclear reaction, by deuteron irradiation of enriched ^60^Ni or even natural nickel targets (McCarthy et al. 1999; Svedjehed et al. 2020). The main advantage of this production route is its affordable target material. However, deuteron beams are not featured in most compact cyclotrons, which may be seen as a drawback. Moreover, the ^61^Ni(p,n)^61^Cu nuclear reaction is the main chosen production route (Szelecsényi et al. 1993; McCarthy et al. 1999), with a high cross section and without co-production of ^60^Cu for proton energies below 15 MeV, but an expensive ^61^Ni enriched target material, due to the low ^61^Ni abundance (IAEA 2023). Another alternative is the ^62^Ni(p,2n)^61^Cu reaction, which we further explore in this work. In this case the target material results cheaper due to the higher ^62^Ni abundancy over ^61^Ni, but the energy window for the reaction is shifted to energies higher than 14 MeV. Alternatively, the ^64^Zn(p,α)^61^Cu nuclear reaction, presents a lower cross section but also a more affordable target material. Solid and liquid, natural zinc and enriched ^64^Zn targets have been explored (Thieme et al. 2013; Szelecsényi et al. 2006; Dellepiane et al. 2022; Fonseca et al. 2022). However, copper purification from zinc targets have proven to be not so straight-forward as separation from nickel targets, the former including multi-step separations and/or pH adjustments (Thieme et al. 2013; Brühlmann et al. 2023).

**Fig. 1:**
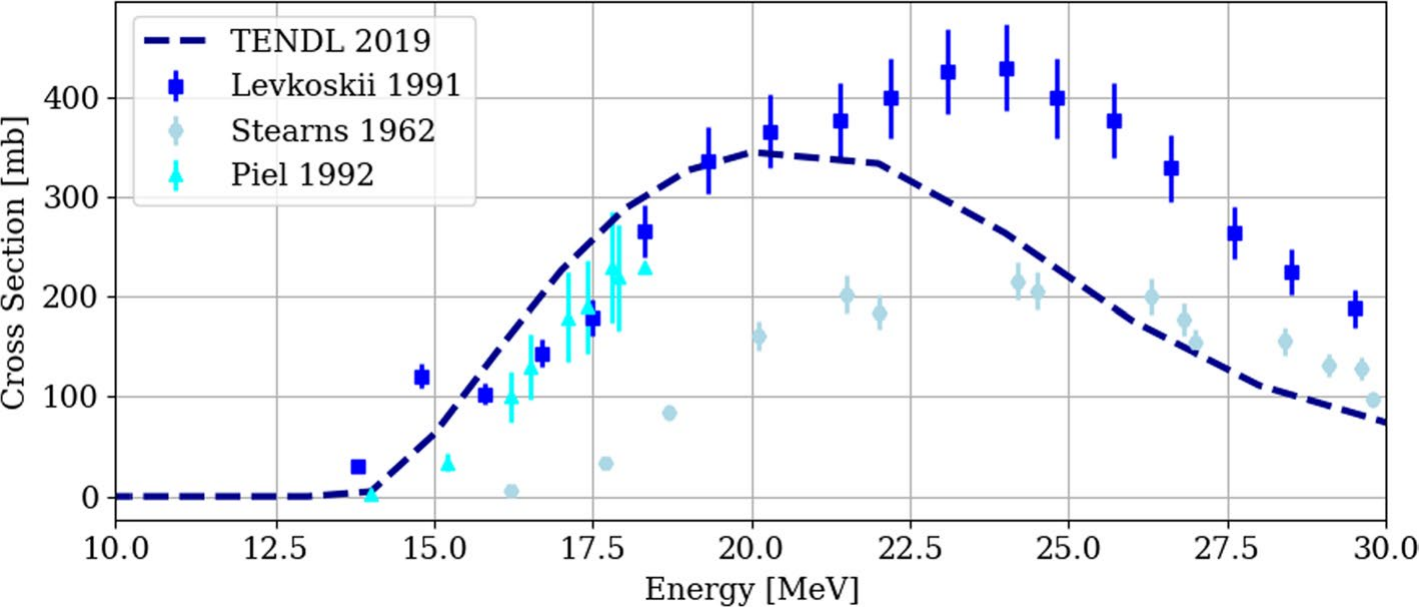
Reported experimental and TENDL simulation cross section for the ^62^Ni(p,2n)^61^Cu reaction

The experimental cross section and the results of the TENDL simulation of the ^62^Ni(p,2n)^61^Cu nuclear reaction are plotted in Fig. 1 (Stearns 1962; Levkovskii 1991; Piel et al. 1992; TENDL 2019). From this figure, substantial dispersion in the results reported from different authors can be seen, which leads to uncertainties on the ^61^Cu yield calculation.

We report in this article the production of ^61^Cu from proton irradiation of electrodeposited enriched ^62^Ni targets via the ^62^Ni(p,2n)^61^Cu nuclear reaction. We discuss the targetry and target radiochemistry developed as well as the product solution characterization, using as a basis the expertise acquired by our group from the nickel-based production of ^64^Cu (Thieme et al. 2012).

## Methods

### Materials

Solutions used consisted of ultrapure 30% hydrochloric acid (Merck KGaA), ultrapure 95% sulfuric acid (Roth GmbH) and ultrapure 20% aqueous ammonia (Roth GmbH) in addition with deionized milli-Q^®^ water. Citric acid monohydrate was purchased from Acros Organics B.V.B.A. (USA) and ammonium acetate 99.999% trace metal basis was acquired from Sigma-Aldrich (USA). Bio-Rad pre-packed Poly-Preps anion exchange columns (0.8 × 4 cm^2^, AG-1 × 8 Cl- form, 200–400 mesh) and 1 mL TrisKem TK201 (pre-packed, 50–100 µm particle size) cartridges were acquired. Gold discs (99.999%, 23 mm diameter, 2 mm thickness) with a centered deepening (10 mm diameter, 0.5 mm depth) were used as substrate for the electrodepositions. Enriched ^62^Ni in the form of metallic ingots was bought from ISOFLEX USA, with isotopic composition (provider specifications) as shown in Table 2.

**Table 2:** Isotopic composition of the ^62^Ni enriched target material, as specified by supplier

Isotope	^58^Ni	^60^Ni	^61^Ni	^62^Ni	^64^Ni
Content [%]	0.01	0.01	0.44	99.36	0.18

### Target preparation

Target preparation consisted of ^62^Ni electrodeposition from [^62^Ni]NiSO_4_ solutions, following the method already standardized by our group for ^64^Ni electrodeposition (Thieme et al. 2012). Basically, fresh enriched ^62^Ni metal ingots (100–105 mg) would be dissolved in 5 mL of 8 M HCl while heating up to 100 °C, and the resulting green solution passed through a 2 mL AG-1 × 8 column in order to remove possible impurities (e.g. stable copper ions). Alternatively, a recycled [^62^Ni]NiCl_2_ fraction (ca. 7 mL, ^62^Ni content 50–95 mg) could be directly used without further purification. This solution would be then evaporated to dryness and re-dissolved in 1 mL 47.5% (w/w) sulfuric acid and neutralized with diluted aqueous ammonia (10% w/w), adjusting to a pH of 9 and obtaining a deep-blue colored solution (ca. 8 mL). This solution was transferred to an electroplating device consisting of a glass vessel with a graphite anode (99.9995% graphite rod from Alfa Aesar, Germany). A gold substrate acted as the cathode, with the electrodeposition taking place in a 7 mm diameter circle. A voltage of 2.7 V was applied overnight (ca. 20 h, starting current 26 mA), leaving afterwards a colorless solution.

### Irradiation parameters optimization

**Fig. 2:**
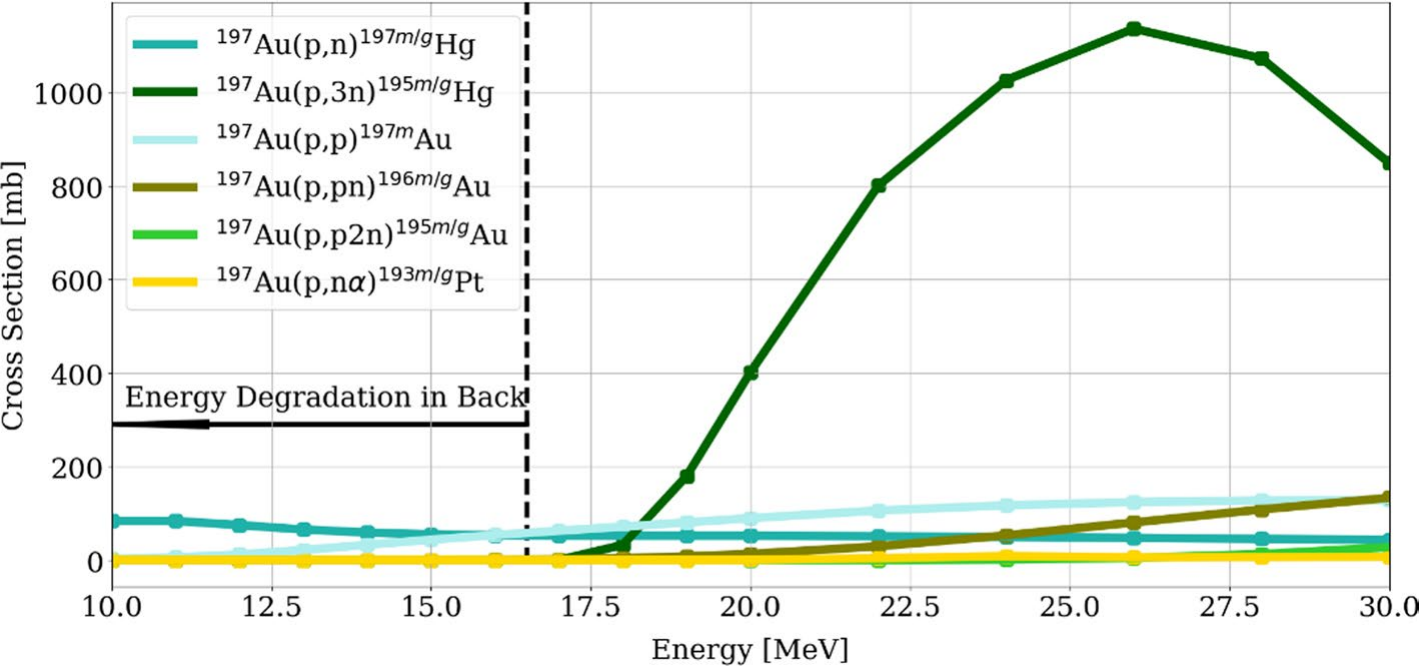
Simulated cross section of relevant nuclear reactions from proton bombardment of a ^197^Au target. In dotted line the desired energy limit (16.5 MeV) to reduce the backing activation

The ^62^Ni(p,2n)^61^Cu nuclear reaction occurs with proton energies higher than 14 MeV, reaching a maximum for energies between 20 and 22 MeV as shown in Fig. 1. Considering the target thickness, the exiting energy from the ^62^Ni target is relevant due to the activation of the backing material, even more when the target backing is reused several times. This factor in combination with chemical resistance for the nickel dissolution and favorable thermal conductivity, led to the election of gold as the target backing. Nevertheless, proton activation of gold is not negligible, and the cross section of relevant nuclear reactions leading to radionuclides from a gold target (i.a. ^197^Au) for energies below 30 MeV can be seen in Fig. 2 (TENDL cross section data TENDL 2019).

Production of ^197 m/g^Hg (half-lives 23.8 h and 64.1 h, respectively) and ^197m^Au (8 s) is inevitable, but the latter results irrelevant due to its short half-life. Nevertheless, higher proton energies result in production first of ^195 m/g^Hg (41.6 h and 10.5 h, respectively), leading to long-lived ^195^Au (186 d), and of ^196 m/g^Au (9.6 h and 6.17 d, respectively). Production of all of the later mentioned radionuclides can be avoided by limiting the proton energy. Further discussion of the backing material activation is presented in the Additional file 1.

**Fig. 3:**
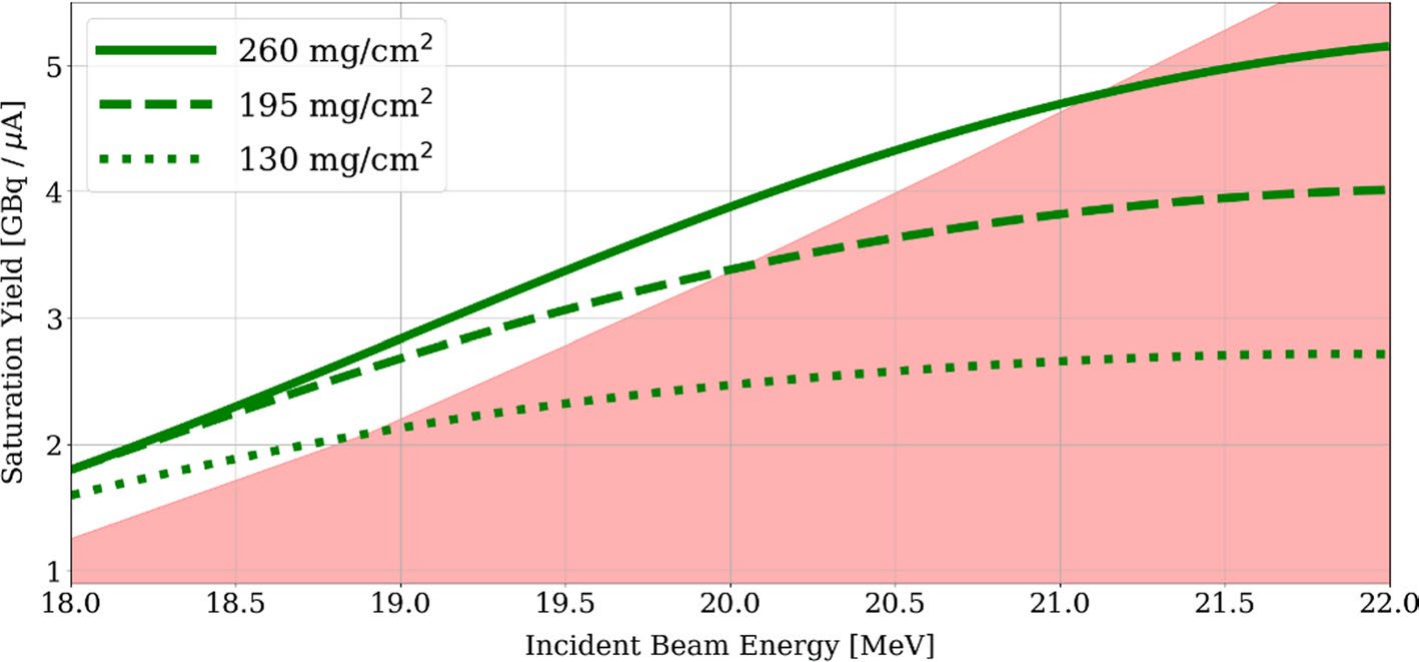
^61^Cu saturation yields for three target thicknesses as a function of the incident proton energy. The red area represents proton incident energies resulting in an exiting energy greater than 16.5 MeV

Moreover, theoretical ^61^Cu saturation yields were calculated exploring different energy windows and considering the exiting energy limitation, with the method previously described by our group (Brühlmann et al. 2023). Due to the relatively good agreement between experimental cross section results and the TENDL simulation for energies below 22 MeV (Fig. 1), the latter data was used. However, it is important to mention that due to the huge differences in the cross section measurements, the values obtained serve only as a first estimation. In Fig. 3 estimated saturation yields for three targets thicknesses, 260 mg/cm^2^, 195 mg/cm^2^ and 130 mg/cm^2^, as a function of the incident proton energy are shown.

### Target irradiation

The target irradiation was carried out using the 90° solid target configuration of the TR-Flex (ACSI—Advanced Cyclotron System Inc) cyclotron installed at the HZDR (Helmholtz-Zentrum Dresden-Rossendorf). The proton beam profile has been previously characterized; a FWHM of 12 mm to 14 mm in an energy range of 14–30 MeV has been quantified (Kreller et al. 2020). Cooling of the target is performed with water on the backside (6 L/min, 20 °C) and with helium at the front (300 L/min, 20–25 °C).

The incident proton energy was modified for each target, aiming not to exceed 16.5 MeV as the exit energy and to avoid high activation of the gold backing. As a simplification, the incident energy of the proton beam could be calculated as a linear function of the target mass with Eq. 1. Since the stopping power is a function of the incident energy, the energy degraded in the target is not linear with the target mass. However, due to the small range of target masses between 50 and 100 mg, the dispersion in the exiting energy would be below 0.1 MeV and regarded as acceptable.

1 $${E}_{in}=19. 8 MeV + 0.044 MeV/mg \bullet {m}_{Target}$$

**Fig. 4:**
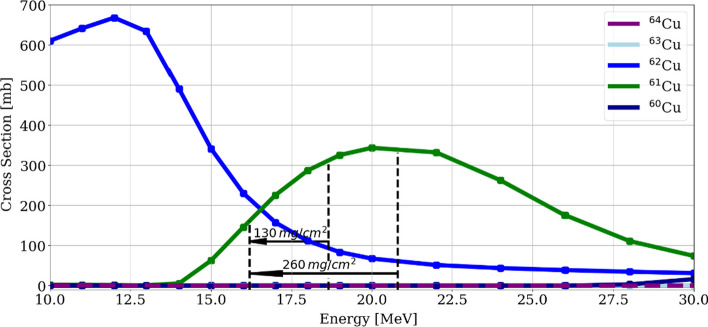
Simulated cross section from TENDL weighted for the ^62^Ni enriched target leading to copper (radio) isotopes

Following the Eq. 1, an extracted proton energy of (22.0 ± 0.1) MeV to (24.0 ± 0.1) MeV from cyclotron was degraded down to (18.6 ± 0.1) MeV to (20.8 ± 0.1) MeV with a 650 µm aluminum degrader in front of the target. In Fig. 4, the cross sections leading to copper radionuclides and stable ^63^Cu, weighted with the target composition from Table 3, as well as the energy degraded within the target are shown. The target was irradiated with a proton current of 70 µA for 1–2 h. In particular, considerable amounts of ^62^Cu is co-produced by proton irradiation of such target, however, assuming end of purification (EOP) three hours after end of bombardment (EOB), a reduction to the kBq range is expected.

**Table 3:** Radionuclide impurities in the [^61^Cu]CuCl_2_ fraction corrected to EOP, using both the 2 mL AG1- × 8 ion exchanger and the 1 mL TK201 resin

Separation column	^62^Cu/%	^64^Cu/%	^57^Co/%	^58^Co/%	^58m^Co^a^/%
AG1- × 8	n/q	< 0.35	< 2E-6	< 6E-4	< 0.05
TK201	n/q	< 0.35	< 1E-6	< 3E-4	< 0.02

^a^Estimated content, not possible to directly quantify

### Radiochemical separation

After the 1-h decay time to reduce the content of short-lived ^62^Cu, the target was washed with 10 mL of a 0.1 M citric acid solution for 15 min to remove the presence of metallic ions, and rinsed 2 times with 10 mL of milli-Q H_2_O. This step could increase the molar activity of the product by removing stable Cu^2+^ ions from the target backing, coming from the cooling water circuit. In the obtained washing, less than 3 MBq of ^61^Cu would be lost (EOP corrected).

**Fig. 5:**
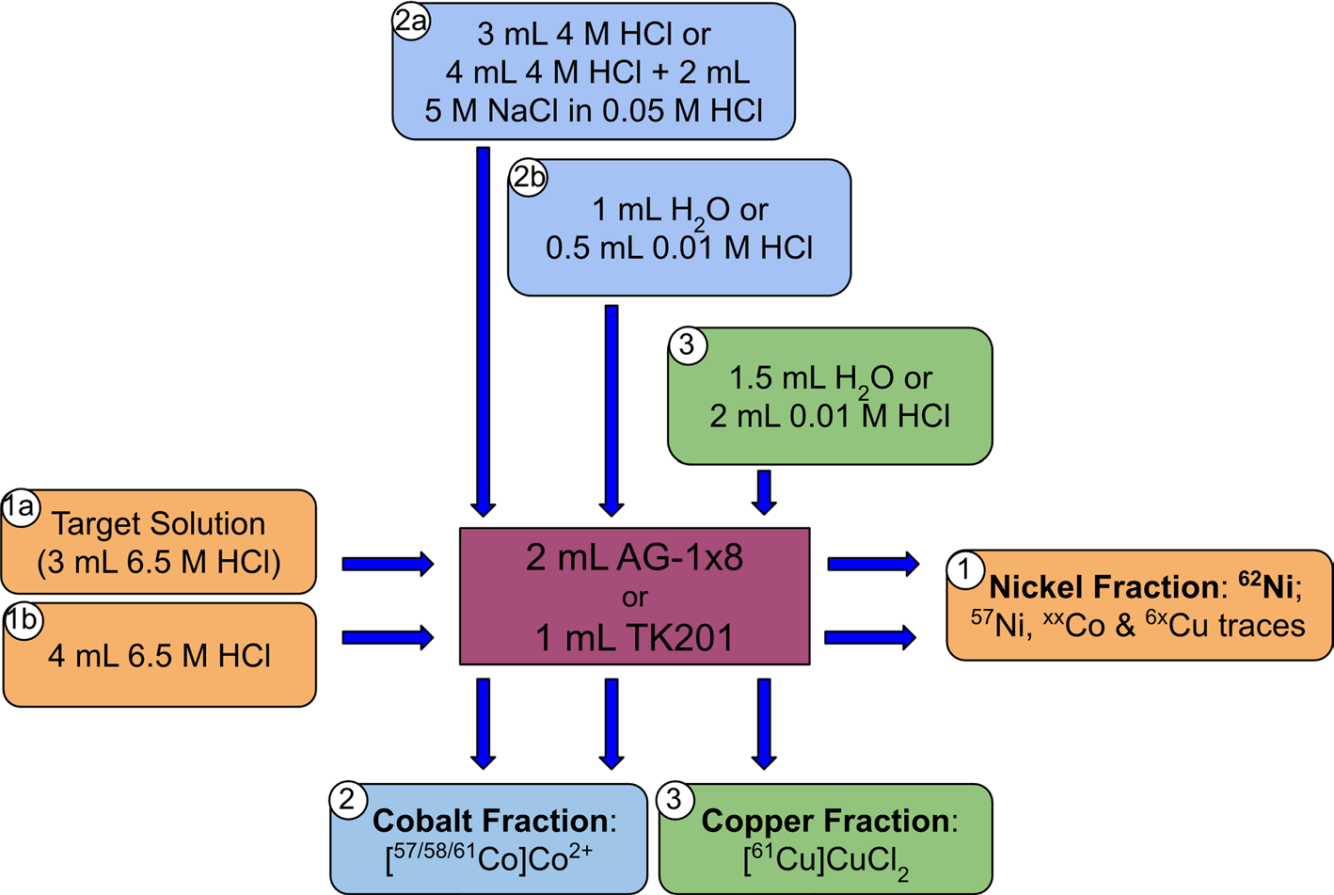
One-step radiochemical separation scheme of the ^61^Cu purification. Three main fractions are obtained: the nickel fraction used for target recovery, the cobalt fraction (waste) and the product [^61^Cu]CuCl_2_ fraction

The enriched ^62^Ni targets were first purged with 3 mL of 6.5 M HCl at room temperature to further reduce the stable metal impurities content. Again, in this step some activity was lost, but represented no more than 50 MBq (EOP corrected). Afterwards, the target would be dissolved in 3 mL of 6.5 M HCl at 90 °C. The purge as well as the target dissolution are performed with a reactor where the whole target (including the backing) is immersed in acid, in a closed system with argon and fume trap. Radiochemical separation consisted of an one-step chromatographic separation with an anion exchanger or an extraction chromatography resin (2 mL AG-1 × 8 or 1 mL TK201, respectively), where the raw target solutions were pumped with a peristaltic pump at a 0.25 mL/min rate. A scheme of the separation is shown in Fig. 5. This raw target solution is loaded (1a) onto the preconditioned (1 × 2 mL H_2_O, 2 × 3 mL 6.5 M HCl) column and washed with 4 mL of 6.5 M HCl (1b) to quantitively recover the ^62^Ni target material. The following steps were differentiated for the two methods.

The anion exchanger column would be washed with 3 mL of 4 M HCl (2a) and 1 mL of H_2_O (2b) to remove the radiocobalt followed by the ^61^Cu elution with 1.5 mL of H_2_O (3). Although the elution was performed with water, the residual HCl in the column produced the radiocopper fraction to be contained in 1.5 M HCl. This [^61^Cu]CuCl_2_ could be used for radiolabeling with a strong buffer or evaporated to dryness and re-dissolved in 700 µL of H_2_O to obtain a more concentrated [^61^Cu]CuCl_2_ solution.

On the other hand, the TK201 cartridge was washed with 4 mL of 4 M HCl and 2 mL of 5 M NaCl in 0.05 M HCl (2a) to remove the radiocobalt impurities, similar as previously described (Svedjehed et al. 2020). A first 0.5 mL of 0.01 M HCl (2b) was used to reduce the chloride concentration of the resin and the [^61^Cu]CuCl_2_ would be eluted in 2 mL of the same solution (3) ready to label.

### Product characterization

Characterization of the nickel, cobalt and copper fractions was performed by high-resolution gamma-spectroscopy using an energy- and efficiency-calibrated Mirion Technologies (Canberra) CryoPulse 5 HPGe detector. From the nickel fraction 200 µL were filled into a tube with calibrated geometry for the gamma spectroscopy measurement. On the other hand, ca. 10 µL from the cobalt fraction or ca. 1 µL of a diluted [^61^Cu]CuCl_2_ product solution (between 200 and 600 kBq) were filled into the same tubes and filled with water to achieve a total volume of 200 µL. The measurements were performed within 2 h after EOP, with a set live-time of 600 s for the three probes and ensuring a dead-time below 5%. In addition, a fourth probe containing 100–200 µL of the [^61^Cu]CuCl_2_ fraction (500–1000 MBq ^61^Cu at EOP, total volume filled with water to 200 µL when needed) was also analyzed between 48 and 72 h after EOP for 1 h live-time to better detect impurities and quantify the radionuclidic purity (RNP) of the product. Activities were automatically calculated with the software Genie2000 (V. 3.4.1).

ICP-MS measurements were performed to quantify the content of stable cations in the product solution. From the copper fraction, ca. 200–400 µL of solution containing approximately 1–2 GBq of ^61^Cu were taken for the measurement. The presence of copper (Cu^2+^), nickel-62 ([^62^Ni]Ni^2+^), cobalt (Co^2+^), aluminum (Al^3+^) and lead (Pb^2+^) was looked into. The Cu^2+^ content is used to determine the molar activity of the product. In addition, lead (Pb^2+^) contamination was also analyzed by this mean. ICP-MS measurements were carried out by VKTA (Radiation Protection, Analytics & Disposal Rossendorf Inc. (VKTA) Dresden, Germany).

Furthermore, the apparent molar activity (AMA) of the product was quantified by titration with the macrocyclic ligand 1,4,8,11-Tetraazacyclotetradecane-1,4,8,11-tetraacetic acid (TETA). Radiolabeling followed the method proposed by Thieme et al. (Thieme et al. 2012). Basically, 5.0–10.0 µL of a diluted [^61^Cu]CuCl_2_ solution (approximately 2 MBq ^61^Cu) was added to aqueous solutions containing different complexing agent concentrations (14–140 nM, 250 µL total volume, 200 mM ammonium acetate buffer pH 6), and mixed for 30 min at room temperature. The TETA complex was performed on an iTLC-SA paper developed with milli-Q water. While the [^61^Cu]CuCl_2_ remains at the start of the paper, the radiometal complex runs to the front. Complexation of over 70% was used to determine the AMA.

## Results

### Target preparation

**Fig. 6:**
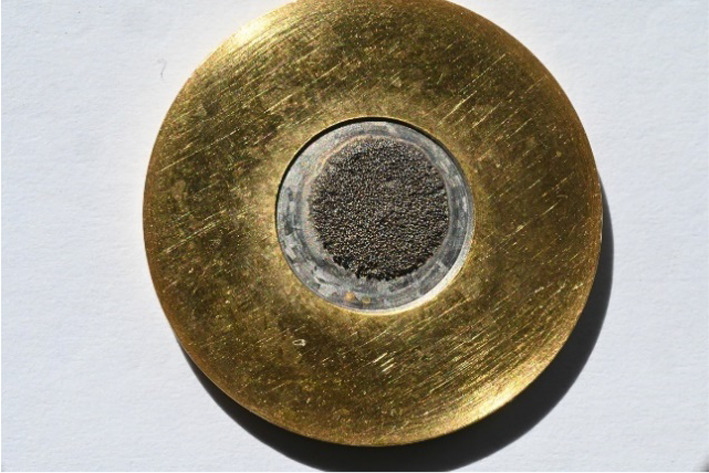
Electrodeposited 70 mg ^62^Ni (182 mg/cm^2^) target onto a gold backing

Target masses in the 50–100 mg range were obtained, accounting for ^62^Ni surface densities between 130 and 260 mg/cm^2^. The electrodeposition yield obtained was mainly over 95% (for the fresh ^62^Ni (97.1 ± 1.4)%, n = 3), while the whole recovery yield from the old target dissolution to the new target electrodeposition amounted over 90%. A typical 182 mg/cm^2 62^Ni target is shown in Fig. 6.

### Activity yields and purification efficiency

The saturation yield of the radioisotope ^61^Cu was in the range of 800–1500 MBq/µA at EOB, depending on the surface density, i.a. ^62^Ni mass. In fact, activities between 10 and 20 GBq at EOB were reached, while after purification between 4 and 8 GBq of a [^61^Cu]CuCl_2_ solution were obtained. Selected irradiation results are available in the Additional file 1. This saturation yield accounts for 25% of the calculated theoretical one presented in Fig. 3.

After purification following the described methods, 65–80% of the ^61^Cu activity (decay corrected) was obtained in the [^61^Cu]CuCl_2_ fraction, with no significative difference between both separation methods. The major activity loss was during the target dissolution, where up to 12% of the radioactivity could not be pumped out of the dissolving reactor, thus making the differences in efficiency of both separation methods negligible. In particular, the separation yields obtained were (84 ± 5) % for n = 4 (AG-1 × 8) and (83 ± 7)% for n = 3 (TK201), decay corrected and considering only the activity of the raw solution and not the reactor loss.

Moreover, when using the anion exchange method, the product would be contained in 1.5 mL of ca. 1.5 M HCl, which could be dried and re-dissolved in water or directly used for radiolabeling with a strong buffer solution. In the case of the TK201 resin, the product solution consisted of 0.01 M HCl ready to label with. The whole purification process, target dissolution inclusive, took about 2 h.

### Radionuclide purity characterization

**Fig. 7:**
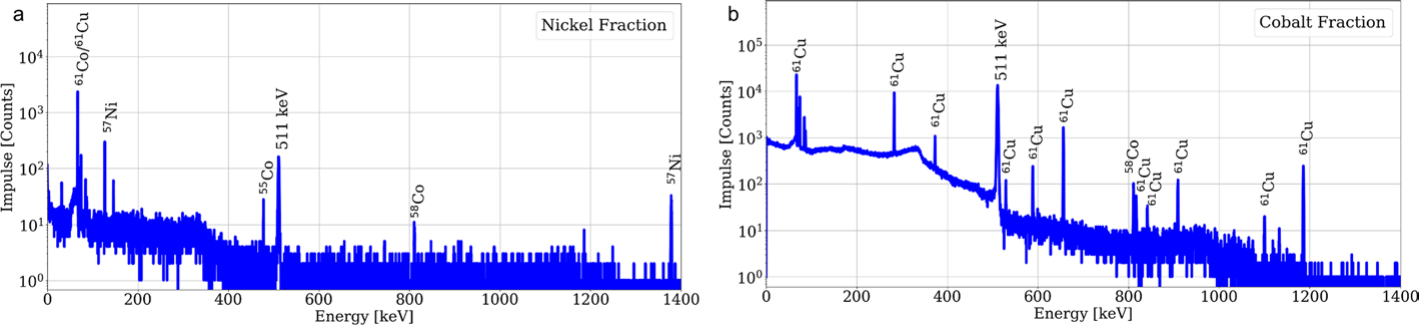
Gammaspectroscopies of the nickel (**a**) and cobalt (**b**) fractions within 1 h after EOP for the AG-1 × 8 based separation. A live-time of 600 s was set with a dead-time below 5%

Gammaspectroscopies of the nickel and cobalt fractions are shown in Fig. 7. In the target material fraction, ^57^Ni was identified accompanied by some minor radiocobalt, while in the cobalt fraction some breakthrough of ^61^Cu was already detected.

**Fig. 8:**
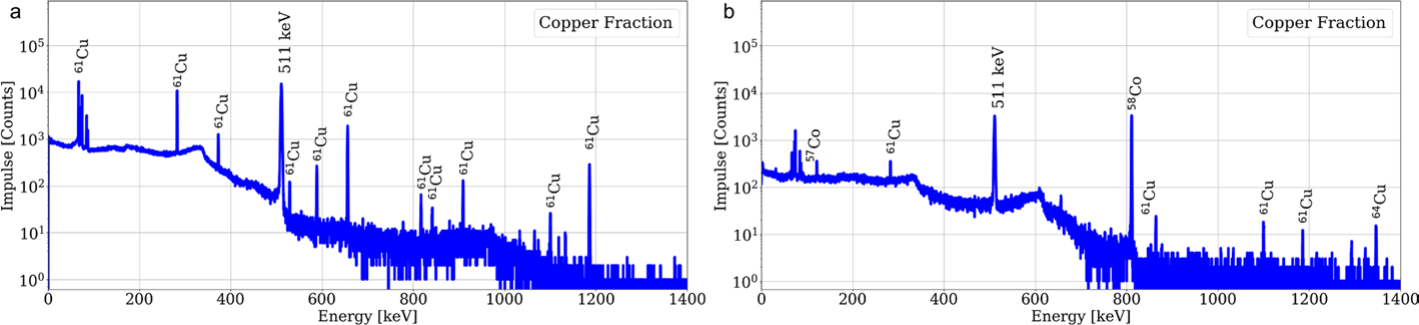
Gammaspectroscopies of the [^61^Cu]CuCl_2_ fraction within 1 h after EOP (**a**) and 70 h after EOP (**b**) for the AG-1 × 8 based separation. A live-time of 600 s and 3600 s was set respectively with a dead-time below 5%

In addition, shortly after purification, no other radionuclide than ^61^Cu could be identified in the product fraction, however, after a decay time of ca. 3 days the presence of radiocobalt and ^64^Cu was quantified. The results of a gammaspectroscopy of the copper fraction performed shortly after EOP and one corresponding to a decayed product fraction are presented in Fig. 8.

Moreover, the RNP of the product fraction was calculated with the quantified radioimpurities from the gammaspectroscopy, resulting in over 99.6% at EOP (over 99.8% at EOB) for both purification methods. The radionuclide impurities are presented in Table 3. It is important to mention, that although ^58m^Co is expected to be present both in the cobalt and the copper fractions, it was not possible to directly quantify the activity of the radionuclide due to the low energy and intensity of its most prominent γ-line (24.9 keV, 0.04%). Thus, an estimation based on the measured ^58g^Co activity and the theoretical yield was performed and is further explained in the Additional file 1.

### Molar activity quantification

Metallic impurity content was analyzed by ICP-MS. These results obtained for the [^61^Cu]CuCl_2_ fractions from both separation methods as well as the ^61^Cu activity concentrations are presented in Table 4.

**Table 4:** ICP-MS results from the [^61^Cu]CuCl_2_ fraction, using both the AG1- × 8 ion exchanger and TK201 resin

Separation column	Activity Conc. (@ EOP)/GBq/mL	Cu^2+^ Conc./ppb	Co^2+^ Conc./ppb	Ni^2+^ Conc./ppb	Al^3+^ Conc./ppb	Pb^2+^ Conc./ppb
AG1- × 8	3.8	590	< 0.04	14,000	< 5.3	31.8
TK201	2.2	235	< 0.016	9700	< 11	21.8

In addition, the molar activity of both product fractions was quantified. For the anion exchange method, a molar activity of 411 GBq/µmol at EOP, i.e. 886 GBq/µmol at EOB was determined, while for the TK201 method molar activities of 605 GBq/µmol and 1280 GBq/µmol at EOP and EOB respectively were calculated.

Moreover, test radiolabeling with the macrocycle TETA proved an AMA between 130 and 260 GBq/µmol at EOP which translates to over 550 GBq/µmol at EOB. In particular, higher AMAs were determined in batches containing higher ^61^Cu activity, regardless of the purification method.

## Discussion

### Activity yields and purification efficiency

The attained experimental saturation yields represent about 25% of the theoretical calculated yield, revealing a larger disagreement than the one observed with other targets irradiated at our cyclotron, as reported in our previous work, where ca. 60% of the theoretical value was achieved (Brühlmann et al. 2022, 2023; Reissig et al. 2020). The uncertainty in the determination of the cross section can be a source of error, where differences of about 50% can be observed between the different measurements. Another critical factor is the uneven nickel electrodeposition in combination with the small cross section of the ^62^Ni(p,2n)^61^Cu nuclear reaction for lower energies; as usual, thinner electrodeposition regions have a lower yield, while thicker electrodeposition areas do not compensate this effect, since the cross section is already too small for lower energies. In fact, the reaction cross section of over 300 mb for 20 MeV is only 150 mb for 16 MeV (calculated exiting energy) and further drops to 50 mb for 15 MeV (Fig. 1). In consequence, thicker electrodeposition areas, where a higher proton energy is degraded, present only a slightly higher ^61^Cu production, while thinner sections, where the energy degradation is lower than expected, the potential production loss is huge.

The decay time after irradiation enabled the reduction in the absorbed dose while performing the radiochemical separation. At EOB, an ^62^Cu activity in the 100 GBq scale is expected, which after 1 h would represent 1.4 GBq and only 2.5 kBq after 3 h, which is about the time between EOB and EOP. Furthermore, from the close to 20 GBq ^61^Cu activity at EOB, after 3 h still over 10 GBq are available, in contrast to the ^62^Cu traces. In consequence, quantification of ^62^Cu in the [^61^Cu]CuCl_2_ product results impossible in practice.

Regarding scalability, a 70 µA, 200-min irradiation (ca. one ^61^Cu half-life) would lead to ^61^Cu activities between 28 and 53 GBq at EOB (depending on the target mass), which followed by an efficient radionuclide purification could lead to activities of up to 20 GBq of [^61^Cu]CuCl_2_ at EOP.

### Radionuclide purity characterization

Based on the results from the gammaspectroscopies, it is possible to conclude that a [^61^Cu]CuCl_2_ product with low content of radionuclide impurities was obtained. Moreover, the product solution would have a RNP of over 99% at least 14 h (over 4 half-lives) after EOP. What is more, since the main radioimpurity quantified was ^64^Cu (accounting for less than 0.35% of the radioactivity at EOP) and this radionuclide is also a β^+^-emitter, the mentioned decrease in the RNP results even less relevant.

### Molar activity quantification

The values obtained for the molar activity and the AMA are in accordance, even more when considering that for the AMA not only the stable copper ions play a role but also other metallic cations in solution. Moreover, slight differences in the pH can also influence the radiochemical yield of the test radiolabeling. Therefore, the AMA is always expected to be lower than the molar activity of the [^61^Cu]CuCl_2_.

Furthermore, the AMA of the produced [^61^Cu]CuCl_2_ results over 40 GBq/µmol for at least 9 h after EOP, which guarantees quantitative radiolabeling by molar activities comparable to that applied in clinical uses with ^68^ Ga, e.g. 35.5 GBq/µmol ^68^ Ga-PSMA-11 (Kleynhans et al. 2020; Lin et al. 2020), with the advantage of ^61^Cu presenting a longer half-life.

One source of stable copper in the solution is the production of ^63^Cu by the ^64^Ni(p,2n)^63^Cu nuclear reaction, however, due to the low ^64^Ni content (0.18%), the impact should be negligible in comparison with external contamination, e.g. from the water-cooling circuit. Since the nickel electrodeposition was performed after purification of the material, either by using recycled target material or fresh nickel pre-purified with an anion exchanger column, this stable copper contribution is also expected to be minimal. Considering these stable copper sources and their impact, it is reasonable to conclude that the copper content is independent of the activity produced and thus the molar activity should increase with production batches of higher ^61^Cu content. Moreover, the stable copper content in the product could be further decreased if the quality of the cooling water circuit would be monitored.

In Table 4 the reported value for ^62^Ni concentrations appear to be quite high. However, considering the whole volume of the product fraction, for the AG-1 × 8 separation method a ^62^Ni content of 21 µg was determined (original target mass 98 mg) and for the TK201 route 19.4 µg was quantified (original target mass 88 mg). These values are in agreement with previously published results which lies in the 5–13 µg range (Thieme et al. 2012; Avila-Rodriguez et al. 2007).

In addition, lead contamination was also observed in the results of the ICP-MS analysis. There are several potential lead sources, since this material provides the shielding of the irradiated targets as well as the product solution. One possibility is contamination of the target with metallic lead while it is being transferred from the cyclotron to the purification module, which would later be dissolved in the 6.5 M HCl. Other alternative is direct contamination of the [^61^Cu]CuCl_2_ fraction, while it is being loaded from the module into the product vials. Since the detected Pb^2+^ is rather low (one order of magnitude lower than Cu^2+^), the value is regarded as acceptable.

## Conclusions

The current article presents a comprehensive production method for the PET radionuclide ^61^Cu. An alternative to the most popular production routes from ^64^Zn and ^61^Ni was studied, reaching for the first time, for the best of our knowledge, activity yields of up to 20 GBq at EOB via the ^62^Ni(p,2n)^61^Cu nuclear reaction. Moreover, two purification methods were studied and lead to similar results regarding the separation yield and the purity of the product. Characterization of the [^61^Cu]CuCl_2_ product showed both high RNP, over 99.6%, as well as high AMA 260 GBq/µmol, EOP corrected. These results lead to conclude that the produced radionuclide could be used up to 9 h after EOP still with high quality. The shorter half-life of ^61^Cu along the higher β^+^ branching ratio can be considered an advantage over ^64^Cu from a dosimetric point of view, thus the availability of this radionuclide plays a key role in the future development of ^61^Cu-based radiopharmaceuticals.

### Supplementary Information

**Additional file 1**. Supplementary Information.

## Data Availability

The datasets used and/or analyzed during the current study are available from the corresponding author on reasonable request.

## Abbreviations

ACSI: Advanced cyclotron system Inc
AMA: Apparent molar activity
EOB: End of bombardment
EOP: End of purification
HZDR: Helmholtz-Zentrum Dresden-Rossendorf
ICP-MS: Inductively coupled plasma mass spectrometry
iTLC: Instant thin layer chromatography
PET: Positron emission tomography
RNP: Radionuclidic purity
SA: Silicic acid
TETA: 1,4,8,11-Tetraazacyclotetradecane-1,4,8,11-tetraacetic
VKTA: Radiation protection, analytics & disposal rossendorf Inc
