# Supplementary material for: Production of the PET radionuclide 61Cu via the 62Ni(p,2n)61Cu nuclear reaction
Source: EJNMMI Radiopharm Chem. 2024 Jan 5;9:3. doi: 10.1186/s41181-023-00233-z (PMC10770005; doi:10.1186/s41181-023-00233-z)
Supplement: Supplementary file 1 — Additional file 1. Supplementary Information. [file 41181_2023_233_MOESM1_ESM.docx]

# Supplementary Information

### Radionuclide Properties

The physical properties of short-lived positron-emitter radionuclides are listed in Table 1 [1,2].

**Table 1:** Physical properties of potential PET radionuclides with half-lives between 1 and 5 hours.

| Radionuclide | Half-Life/h | E_β-,mean_/keV (Intensity/%) | Eγ/keV  (Intensity/%) |
| --- | --- | --- | --- |
| **^43^Sc** | 3.89 | 508 (70.9) 344.5 (17.2) | 372.9 (22.5) |
| **^44^Sc** | 3.97 | 632 (94.27) | 1157 (99.9) |
| **^45^Ti** | 3.08 | 439 (84.8) | No γ-line >1 % |
| **^61^Cu** | 3.34 | 524 (51) 399 (5.8) 238 (2.5) 494 (2.1) | 282.9 (12.7) 656.0 (10.4) 67.41 (4.0) 1185 (3.6) i.a. |
| **^68^Ga** | 1.13 | 836 (87.72) 352.6 (1.19) | 1077 (3.22) |
| **^132^La** | 4.80 | 1454 (14) 1191 (11) 1665 (9.2) 496 (2) 582 (1.4) i.a. | 464.5 (76) 567.1 (15.7) 1909 (9.0) 663.0 (9.0) 1031 (7.8) i.a. |
| **^133^La** | 3.91 | 463 (7.1) | 278.8 (2.44) 302.4 (1.61) 290.1 (1.38) i.a. |

### Backing Activation Analysis

Using the Isotopia tool [3], it was calculated the activities reached of the most relevant radionuclides for a 1-hour, 70 µA proton irradiation of the gold backing for different incident energies. The results are presented in Table 2. Following these results and considering the uniformities of the electrodeposition, an upper limit of 16.5 MeV was stablished for the exiting beam energy to further limit the activation of the gold backing.

**Table 2:** Calculated activities at the gold backing for a 1-hour 70 µA proton irradiation and different incident energies.

| Incident Energy at Au back | Activity ^197m^Hg /MBq @ EOB | Activity ^197g^Hg /MBq @ EOB | Activity ^195m^Hg /MBq @ EOB | Activity ^195g^Hg /MBq @ EOB |
| --- | --- | --- | --- | --- |
| 15 | 590 | 230 | - | - |
| 17.5 | 840 | 300 | - | - |
| 20 | 1100 | 360 | 220 | 1000 |

### Irradiation Parameters

**Table 3:** Activity yields estimated for a 1-hour 70 µA proton irradiation and different incident energies. Two target thicknesses are considered, 100 mg (ca. 260 mg/cm^2^) and 50 mg (ca. 130 mg/cm^2^).

| Incident Proton Energy /MeV | Exiting Proton  Energy /MeV | Activity ^61^Cu @EOB  /GBq | Activity ^62^Cu @EOB /GBq |
| --- | --- | --- | --- |
| 19 | 13.7 – 16.5 | 37 – 28 | 306 – 110 |
| 20 | 15.0 – 17.6 | 51 – 33 | 239 – 86 |
| 21 | 16.2 – 18.6 | 63 – 36 | 185 – 67 |
| 22 | 17.4 – 19.8 | 69 – 36 | 143 – 53 |

## Activity Yields

Some representative target irradiation results are presented in Table 4.

**Table 4:** Irradiation conditions and results of selected targets.

| Target Mass /mg | Proton Energy /MeV | Current /µA | Irradiation Time /min | Raw Activity @EOB /GBq | Prod. Activity @EOP /GBq | Sat. Yield /MBq/µA |
| --- | --- | --- | --- | --- | --- | --- |
| 100 | 18.8 | 70 | 60 | 12.5 | 3.5 | 955 |
| 101 | 20.8 | 70 | 60 | 20.0 | 6.5 | 1560 |
| 88 | 20 | 70 | 60 | 17.2 | 5.4 | 1310 |
| 67 | 19.3 | 70 | 60 | 10.6 | 4.3 | 800 |

### Radionuclidic Purity and ^58m^Co Estimation

Since ^58m^Co results extremely difficult to measure due to its decay characteristics (internal transition 100 %, most prominent γ-line 24.9 keV, 0.04 %), an estimation of its activity was performed.

Assuming a 1 hour, 70 µA and 20.8 MeV proton irradiation of a 100 mg ^62^Ni target, theoretical activities of 535 MBq and 4.24 MBq for ^58m^Co and ^58g^Co respectively are calculated [3]. When a decay time of 2 days or longer is waited (time between EOP and RNP quantification), over 98 % of the ^58m^Co would have decayed to the ground state contributing to the total ^58g^Co activity with a factor equal to the ratio between their half-lives (9.1 h and 70.86 d, respectively [1]). Moreover, a theoretical ^58g^Co activity of 7.02 MBq would be reached two days after EOB, from which only 60 % can be attributed to the original ^58g^Co activity and the other 40 % to the decayed ^58m^Co.

When performing the gammaspectroscopy of the product, it is not possible to differentiate the original ^58g^Co, which is directly produced in the irradiation, and the ^58g^Co coming from the metastable state. However, the ^58m^Co can be then estimated using the ratio of produced ^58m^Co and total ^58g^Co after two days (535 MBq and 7.02 MBq, respectively), thus obtaining a factor 76.2 ^58m^Co/^58g^Co measured.

Then the ^58g^Co impurity at EOP can be quantified using the measured ^58g^Co activity thus being a conservative estimation since only ca. 60 % corresponds to this activity, while the ^58m^Co impurity can be assessed by applying the correction factor to the measured activity.

## Literature

1. IAEA Nuclear Data Services. IAEA, Vienna. 2007-2023. https://www-nds.iaea.org. Accessed 21 March 2023.
2. Brühlmann S, Kreller M, Pietzsch H-J, Kopka K, Mamat C, Walther M, Reissig F (2022) Efficient Production of the PET Radionuclide ^133^La for Theranostic Purposes in Targeted Alpha Therapy Using the ^134^Ba(p,2n)^133^La Reaction. Pharmaceuticals 15:1167.
3. Isotopia - Medical isotope browser. IAEA – Nuclear Data Section, Vienna. 2009-2023. https://www-nds.iaea.org/relnsd/isotopia/isotopia.html. Accessed 27 March 2023.
